# Melatonin and Mitochondrial Redox Homeostasis in Reproduction: Mechanistic Links Between Circadian Signaling and Fertility Outcomes

**DOI:** 10.3390/biology15131000

**Published:** 2026-06-25

**Authors:** Sofoklis Stavros, Panagiotis Christopoulos, Stefanos Dafopoulos, Chrysi Christodoulaki, Efthalia Moustakli, Anastasios Potiris, Maria Tzeli, Athanasios Zikopoulos, Konstantinos Dafopoulos, Peter Drakakis

**Affiliations:** 1 Third Department of Obstetrics and Gynecology, University General Hospital “ATTIKON”, Medical School, National and Kapodistrian University of Athens, 12462 Athens, Greece; sfstavrou@med.uoa.gr (S.S.); pdrakakis@hotmail.com (P.D.); 2 Second Department of Obstetrics and Gynecology, “Aretaieion” Hospital, Medical School, National and Kapodistrian University of Athens, 11528 Athens, Greece; panchrist@med.uoa.gr; 3 University General Hospital of Patras, University of Patras, 26504 Patras, Greece; stefanosntf2001@gmail.com; 4 Department of Obstetrics and Gynecology, General Hospital of Chania, 73300 Chania, Greece; christodoulakichr@hotmail.com; 5 Department of Nursing, School of Health Sciences, University of Ioannina, 45500 Ioannina, Greece; 6 Department of Midwifery, Faculty of Health and Caring Sciences, University of West Attica, 12243 Athens, Greece; mtzeli@uniwa.gr; 7 Department of Reproductive Medicine and Surgery, University College London Hospitals NHS Foundation Trust, 235 Euston Road, London NW1 2BU, UK; thanzik92@gmail.com; 8 Department of Obstetrics and Gynaecology, Faculty of Medicine, School of Health Sciences, University of Thessaly, 41110 Larissa, Greece; kdafop@uth.gr

**Keywords:** redox signaling, mitochondrial bioenergetics, antioxidant defense, gamete quality, embryo development, chronobiology, oxidative balance, Nrf2 pathway, cellular metabolism, reproductive health

## Abstract

The pineal gland regulates circadian physiology through the periodic production of melatonin (MLT). In addition to its established role as a chronobiotic agent, MLT regulates redox homeostasis and mitochondrial physiology. Mitochondria and redox-active molecules, particularly reactive oxygen species (ROS), play essential roles in reproduction, including gamete physiology, fertilization, and early embryonic development. Although excessive oxidative stress (OS) impairs fertility, controlled ROS signaling is necessary for normal reproductive function. This comprehensive review synthesizes current evidence regarding MLT as a key intermediary linking circadian signaling with mitochondrial physiology and redox homeostasis. We discuss molecular pathways through which MLT regulates mitochondrial function, including activation of the Nrf2 signaling pathway, modulation of mitochondrial permeability transition, regulation of electron transport chain (ETC) efficiency, and apoptotic signaling. Furthermore, this study investigates MLT’s ability to scavenge free radicals and activate antioxidant defense mechanisms. Moreover, we review novel findings regarding the effects of MLT in experimental animals and humans, assisted reproductive technologies (ART) such as in vitro fertilization (IVF), and consider the translational significance of the hormone as an enhancer of fertility. We also highlight gaps in the literature, including methodological inconsistencies, supraphysiologic doses, and insufficient data from large human cohorts. Lastly, we discuss an integrative model whereby MLT may function as an important regulator of mitochondrial redox balance, with potential implications for reproductive physiology and reproductive outcomes, and propose new avenues for investigation.

## Simple Summary

The biological clock helps coordinate many functions in the human body, including reproduction. Melatonin, a hormone produced mainly at night, is best known for regulating sleep and daily biological rhythms. However, growing evidence suggests that melatonin also helps protect cells from oxidative damage and supports the function of mitochondria, the structures responsible for cellular energy production. These processes are particularly important in reproductive cells such as sperm, oocytes, and early embryos, which require high levels of energy and are vulnerable to oxidative stress. This review summarizes current evidence regarding the effects of melatonin on mitochondrial function, cellular redox balance, and reproductive health. Evidence from laboratory experiments, animal studies, and clinical research is discussed to highlight both the potential benefits and the current limitations of melatonin use in reproductive medicine. Although melatonin has shown promising effects, evidence regarding major clinical outcomes remains limited. A more complete comprehension of the connection between circadian rhythm biology and mitochondrial biology can provide new insights for future investigation.

## 1. Introduction

The temporal organization of biological systems is vital, where circadian rhythms coordinate biological processes with respect to predictable light–dark environmental conditions [1,2]. Maintaining homeostasis is particularly important for individuals with highly regulated energy metabolism [3]. Melatonin (MLT) is a hormone that mediates circadian signaling through rhythmic synthesis in the pineal gland, primarily at night. MLT is a well-established regulator of the sleep–wake cycle and is now recognized as a pleiotropic regulator of metabolism and mitochondrial bioenergetics [4,5].

Reproductive physiology is an area where circadian biology and bioenergetics interact in a particularly delicate way. Coordination of energy generation, redox signaling, and apoptosis control is essential for gametogenesis, fertilization, and early embryogenesis [6,7]. Alterations in circadian rhythms, which can be caused by environmental, behavioral, and pathological influences, have been shown to contribute to reproductive dysfunction. In this context, MLT is no longer considered only a hormone secreted by the circadian system but also a molecule that interprets time signals into cellular action within the reproductive system [8,9,10].

The mitochondria play a pivotal role in the process. Not only do the mitochondria act as ATP generators, but they also regulate apoptosis signaling pathways, calcium homeostasis, and reactive oxygen species (ROS) generation. The efficacy of the mitochondria is essential in germ cells since the germ cells need a considerable amount of energy and are prone to oxidative stress (OS) [11,12,13]. It has been determined that ROS exerts dual effects in reproductive physiology, where excessive ROS levels contribute to oxidative damage and infertility, whereas moderate ROS levels are essential for physiological processes such as sperm capacitation and oocyte maturation [14].

Recent research has indicated that MLT has a major influence on this mechanism. Apart from its direct antioxidant action against free radicals, MLT is involved in the regulation of mitochondrial energy metabolism, enhances electron transport efficiency, and influences permeability transition and apoptotic pathways [15]. Crucially, MLT is able to induce activation of the endogenous antioxidant defense system, such as by means of the Nrf2 pathway. Hence, MLT is not merely an antioxidant but also a chronobiologically controlled regulator of mitochondrial activity and oxidative-reductive balance [5,16].

Although substantial progress has been made, mechanistic coherence between circadian signaling, mitochondrial function, and reproductive success remains limited. This limitation arises from research that often examines these factors independently, resulting in an incomplete understanding of how temporal information is integrated within mitochondria and may influence reproductive processes [17,18,19]. In addition, translational evidence is inconsistent due to variations in methodology and different parameters evaluated in both human and assisted reproductive studies [17].

**Figure 1:**
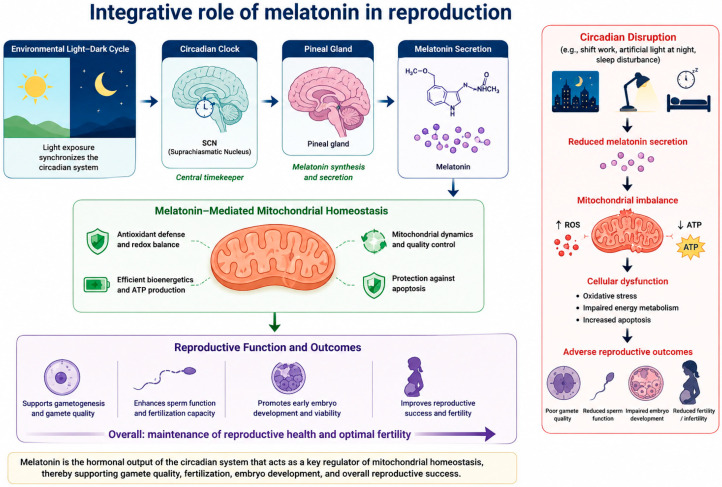
Integrative role of melatonin in reproduction. Circadian rhythms regulate melatonin secretion by the pineal gland, linking environmental light–dark cycles with mitochondrial homeostasis. Through its effects on bioenergetics, redox balance, and cellular survival, melatonin supports gamete quality, embryo development, and reproductive success, whereas circadian disruption may contribute to mitochondrial dysfunction and impaired fertility.

Previous reviews have discussed melatonin as an antioxidant or as a regulator of reproductive physiology; however, these topics are frequently examined separately. The novelty of the present review lies in the integration of circadian signaling, mitochondrial regulation, redox homeostasis, and reproductive outcomes into a unified conceptual framework. By focusing on melatonin as a mechanistic bridge between temporal biological signaling and mitochondrial function, this review provides a broader perspective on the role of melatonin in reproductive health. Within the scope of this review, we hypothesize that MLT plays a role as an integrator that links circadian biology with mitochondrial redox regulation within reproductive systems. This study aims to integrate current knowledge of the molecular pathways through which MLT modulates mitochondrial energy metabolism, oxidative status, and apoptosis, thereby influencing gamete quality and embryo survival. By integrating data from cell culture experiments, animal models, and clinical observations, this study defines a conceptual model of MLT as a time-dependent regulator of reproductive function and identifies key areas for future research. The integrative relationship between circadian signaling, MLT, mitochondrial regulation, and reproductive outcomes is summarized in Figure 1.

## 2. Search Strategy and Evidence Selection

This review was conducted as a narrative review of the literature examining the role of melatonin in reproductive biology, with particular emphasis on circadian regulation, mitochondrial function, redox homeostasis, and assisted reproductive technologies (ART). Literature searches were performed in PubMed, Scopus, and Google Scholar using combinations of the following keywords: “melatonin”, “circadian rhythm”, “pineal gland”, “mitochondria”, “oxidative stress”, “redox homeostasis”, “Nrf2”, “SIRT3”, “reproduction”, “fertility”, “gametes”, “oocyte”, “sperm”, “embryo development”, and “assisted reproductive technology”.

Priority was given to peer-reviewed articles published in English, including original research studies, clinical investigations, and relevant review articles. Both experimental and human studies were considered to provide a comprehensive overview of current knowledge. Seminal publications were included where necessary to provide historical context, while recent studies were preferentially selected to reflect current advances in the field. Articles not directly related to reproductive biology, melatonin signaling, mitochondrial regulation, or OS were excluded from detailed discussion.

The final selection of studies was based on their relevance to the objectives of the review and their contribution to understanding the relationship between melatonin, circadian biology, mitochondrial function, redox regulation, and reproductive outcomes.

## 3. Mitochondrial Function and Redox Homeostasis in Reproductive Biology

### 3.1. Mitochondrial Function in Gametes and Early Embryos

Mitochondria play a crucial regulatory role in cell biology, extending beyond ATP synthesis to regulate apoptosis, calcium balance, and metabolism. In reproductive biology, mitochondrial efficiency is a major determinant of normal gamete development [20]. It should be noted that the oocyte contains a great number of mitochondria that provide the necessary energy for the maturation process, fertilization, and early stages of embryo development [21,22].

Mitochondria in spermatozoa are highly specialized structures located in the midpiece region, where they synthesize ATP to power sperm motility and fertilization competence [23,24]. Mitochondrial impairment in spermatocytes leads to poor motility, defective capacitation, and DNA damage [25]. In early embryos, mitochondrial energy metabolism is highly regulated because both excessive and insufficient energy production may interfere with development. It is imperative to note that mitochondrial DNA and efficient oxidative phosphorylation play crucial roles in normal embryonic development [26,27,28].

Overall, these findings illustrate the importance of mitochondria’s role as not just being supportive but also instructional to the process of reproduction, influencing cellular decision-making from gametogenesis to early embryogenesis.

### 3.2. ROS and Redox Signaling in Reproduction

A characteristic of mitochondrial function is the synthesis of ROS. The latter are typically known to be harmful compounds produced during metabolism as by-products of electron transport chains (ETCs) [29,30]. However, recent research suggests that ROS play an important role in regulating numerous physiological processes. In reproductive processes, ROS are essential for cellular function and regulate sperm capacitation, the acrosome reaction, and oocyte maturation [31,32,33].

Nevertheless, the favorable impact of ROS is very concentration-specific. Whenever ROS concentrations surpass the cell’s ability to neutralize them, OS ensues, resulting in lipoperoxidation, proteolysis, and even DNA damage. Germ cells are prone to OS due to increased metabolism, the absence of antioxidants at certain stages, and, in spermatozoa, the lack of cytoplasmic repair enzymes [34,35].

Consequently, successful reproduction requires redox homeostasis, which is described as the equilibrium between the synthesis of ROS and the cell’s defense against them. In this way, the functions of ROS will be achieved without damaging the cell itself.

### 3.3. OS and Reproductive Dysfunction

Redox imbalance plays an important role in the development of reproductive disorders [36,37]. For instance, OS in oocytes is known to interfere with mitochondrial location, alter meiotic spindle shape, and reduce the likelihood of embryo formation. In spermatozoa, OS presents as increased DNA fragmentation, decreased motility, and damage to the plasma membrane [38,39].

OS can inhibit cell division and induce changes in the pattern of gene expression in developing embryonic cells. Additionally, mitochondrial failure and OS are related [40,41]. Consequently, a self-amplification loop is formed, in which mitochondrial dysfunction leads to the excessive production of ROS, thereby damaging the cells.

There have been instances where OS has been linked to numerous medical conditions, such as infertility, poor function of ART, and aging-induced reproductive dysfunction. These findings underscore the need for efficient regulation of mitochondrial activity and redox homeostasis within the reproductive organs [38,42].

Due to the impact of mitochondrial activity and redox homeostasis on reproduction, much interest has been generated in studying natural agents capable of controlling these two parameters. The natural compound of choice in such studies is MLT, due to its ability to integrate circadian rhythms with mitochondrial activity and redox homeostasis [43,44]. The importance of mitochondrial activity and redox homeostasis in reproduction is outlined in Table 1 below.

## 4. MLT as a Regulator of Mitochondrial Function

### 4.1. MLT and Mitochondrial Bioenergetics

In addition to its role as a circadian hormone, MLT has emerged as an important regulator of mitochondrial bioenergetics [15,45]. The primary source of ATP is mitochondria through oxidative phosphorylation, which depends on the ETC operating properly. Disruption of ETC function may lead to electron leakage, reduced ATP production, and excessive generation of ROS [29,46,47].

MLT can enhance the efficiency of mitochondria through the stabilization of ETC functioning and decreasing electron leakage. According to experimental research, MLT increases the activity of mitochondrial respiratory chains, which increases ATP generation and minimizes oxidative products [48,49,50]. Regulation of the activity of mitochondria by means of MLT can play a special role in reproduction cells, where the functional activity is closely connected with energy [51]. Moreover, MLT has an impact on the state of mitochondrial membrane potential, which is one of the key factors of proper mitochondrion functioning [5,45].

### 4.2. Regulation of Mitochondrial Dynamics and Apoptosis

The regulation of mitochondrial structure, distribution, and function relies on mitochondrial dynamics, which include processes such as mitochondrial fusion and fission. These processes are fundamental for the degradation of dysfunctional mitochondria and for adjusting their activity to metabolic conditions [52,53]. Mitochondrial dysfunction has been linked to reduced oocyte quality and impaired embryo development. The dynamics of the mitochondria can be controlled through the regulation of specific protein activities related to fusion and fission processes [54,55]. By regulating the fusion–fission processes, MLT could possibly aid in maintaining the structural integrity of the mitochondria while efficiently distributing energy among them [5,43].

Additionally, mitochondria also have an important role in intrinsic apoptosis. This is because this process is highly associated with the maintenance of mitochondrial membrane integrity and the balance between pro- and anti-apoptotic proteins, such as Bcl-2 family members [56,57,58]. MLT prevents cytochrome c release, increases mitochondrial membrane integrity, and regulates apoptotic protein expression, resulting in its anti-apoptotic actions. Moreover, research has shown that MLT might even participate in mitochondrial quality control processes, such as mitophagy, although the exact pathway is not yet fully known [5,59].

### 4.3. MLT and Mitochondrial Redox Regulation

A distinctive feature of MLT is its ability to regulate mitochondrial redox balance through multiple complementary mechanisms. ROS are generated as by-products of mitochondrial respiration and require tight regulation to prevent cellular injury. MLT acts as both a direct and indirect regulator of ROS homeostasis [15,43,49].

MLT acts directly by scavenging various forms of radicals, such as hydroxyl radicals, superoxide anions, and peroxynitrite [60,61]. Unlike many conventional antioxidants, MLT and its metabolites may participate in sequential radical-scavenging reactions that further enhance antioxidant capacity [62,63].

MLT indirectly enhances endogenous antioxidant defenses through activation of redox-sensitive signaling pathways. Among these, the Nrf2 pathway represents one of the most extensively studied mechanisms and is discussed in greater detail in Section 5.2. Through activation of antioxidant defense systems, MLT contributes to the maintenance of mitochondrial redox homeostasis and cellular protection against oxidative stress [64,65,66].

Furthermore, MLT contributes to the regulation of redox signaling in addition to protecting against OS. Maintenance of ROS within physiological ranges allows redox-sensitive signaling pathways required for normal reproductive function to occur without triggering excessive cellular stress responses [44,67].

### 4.4. Integration of Circadian Signaling with Mitochondrial Function

One of the most distinctive properties of MLT is its role in linking circadian timekeeping with mitochondrial function. As a hormone with secretion regulated by the light–dark rhythm, MLT transfers temporal information into the reproductive tissues and other peripheral target cells. Temporal signaling is increasingly recognized as an important regulator of mitochondrial metabolism and cellular homeostasis [5,68].

Mitochondrial activity exhibits circadian oscillations associated with energy metabolism, oxidative phosphorylation, and ROS generation. Synchronization of mitochondrial activity with circadian rhythms may help maintain energy balance and redox homeostasis in accordance with physiological demands [69,70]. Since many reproductive processes, including ovulation and fertilization, are regulated by circadian rhythms, temporal synchronization may be particularly important for reproductive function [6,71].

Circadian disruption may contribute to mitochondrial dysfunction, OS, and impaired reproductive performance. Here, MLT may play a protective role, providing proper temporal regulation [9,72,73].

Collectively, these pathways integrate bioenergetic, redox, and circadian signals, positioning MLT as a central regulator of mitochondrial function. In reproductive systems, where mitochondrial activity directly influences gametogenesis and embryogenesis, this coordinated regulation may be particularly important for maintaining reproductive competence [5]. The physiological impacts of MLT on the mitochondrial pathway are shown in Table 2.

## 5. Molecular Mechanisms Underlying MLT-Mediated Mitochondrial Regulation

### 5.1. Receptor-Dependent and Receptor-Independent Actions

The action of MLT on living cells is brought about by means of receptor-mediated and non-receptor-mediated mechanisms. MLT acts on G-protein coupled receptors such as MT1 and MT2, which are found in reproductive organs like the testes and ovaries [74,75,76]. Activation of the MT1 and MT2 receptors results in the modulation of signaling processes, which include cyclic AMP production, phosphorylation of proteins, and gene transcription. There is some research indicating that MLT receptors might play roles in mitochondrial signaling processes, though more information is needed in this regard [77,78].

However, MLT possesses receptor-independent effects as a result of its amphiphilic nature, enabling its penetration through biological membranes into mitochondria [5,79]. In addition, mitochondria have been proposed as a site of melatonin synthesis, suggesting a direct role of this indoleamine in the regulation of mitochondrial physiology and signaling [80]. In reproductive tissues, mitochondrial melatonin may originate from both circulating pineal-derived melatonin and local extra-pineal synthesis, although the relative contribution of each source during gametogenesis remains incompletely understood. This enables MLT to interact with mitochondrial membranes and components of the ETC, thereby influencing mitochondrial function independently of receptor activation [81].

### 5.2. Regulation of Antioxidant Pathways

MLT regulates cellular antioxidant defenses through activation of several redox-sensitive signaling pathways and through its well-established antioxidant properties, among which the Nrf2 pathway is one of the most extensively studied mechanisms [16,64,82,83].

Experimental studies have demonstrated that MLT enhances Nrf2 activation, resulting in increased expression of antioxidant enzymes including catalase, glutathione peroxidase, and superoxide dismutase [15,84,85,86]. Through this mechanism, MLT strengthens endogenous antioxidant capacity and supports mitochondrial homeostasis. In addition to melatonin itself, its metabolites AFMK (N^1^-acetyl-N^2^-formyl-5-methoxykynuramine) and AMK (N^1^-acetyl-5-methoxykynuramine) contribute to the antioxidant cascade of melatonin. These metabolites retain antioxidant and anti-inflammatory properties and participate in the scavenging of reactive oxygen and nitrogen species [87,88]. However, their specific roles in reproductive physiology and mitochondrial regulation remain incompletely understood and require further investigation.

Beyond Nrf2 signaling, MLT may also influence pathways involved in inflammation, cellular adaptation to stress, and mitochondrial resilience, further contributing to the regulation of redox-sensitive cellular responses [89,90].

### 5.3. Modulation of Mitochondrial Bioenergetic Enzymes

MLT regulates mitochondria enzymes participating in cellular metabolism as well. According to experimental studies, MLT increases the efficiency of ETC complexes I and IV, which positively influences oxidative phosphorylation and prevents electrons from leaking [15,48,49,50,91].

Another target for MLT is the deacetylase in the mitochondria called SIRT3, whose main role includes regulating mitochondrial protein functions and metabolic processes such as oxidation. Activation of SIRT3 leads to mitochondrial deacetylation, especially of those proteins that have a part to play in the antioxidant mechanism and energy metabolism. Studies have shown that there is an increase in SIRT3 activity by the action of MLT, which ultimately leads to decreased levels of OS [92,93,94].

### 5.4. Regulation of Apoptotic Signaling Pathways

The function of MLT is also critical for regulating mitochondria-mediated apoptotic signaling. Apoptotic signaling is regulated by the interaction of pro-apoptotic and anti-apoptotic proteins of the Bcl-2 family, regulating mitochondrial permeability and cytochrome c release [59,95].

MLT modulates apoptotic signaling by suppressing pro-apoptotic factors such as Bax while enhancing expression of anti-apoptotic proteins including Bcl-2. Through regulation of the Bax/Bcl-2 balance, MLT helps preserve mitochondrial membrane integrity and limit cytochrome c release [15,96,97,98].

Additionally, MLT could help to maintain the integrity of mitochondrial membranes and prevent apoptosis by inhibiting the opening of mitochondrial pores. This action would be especially critical in reproductive cells since apoptosis may affect their viability [99].

### 5.5. Integration of Molecular Mechanisms

Importantly, these molecular mechanisms operate as an interconnected regulatory network rather than as isolated pathways. Receptor-mediated signaling, antioxidant responses, bioenergetic regulation, and apoptosis control collectively contribute to the maintenance of mitochondrial homeostasis and cellular adaptation to physiological stress [100,101,102].

Through coordinated regulation of these processes, MLT integrates metabolic, redox, and survival signaling pathways that are essential for reproductive cell function. In reproductive systems, where mitochondrial activity is tightly linked to gamete competence and early embryonic development, this coordinated regulation may be particularly important [5,103]. Molecular mechanisms underlying MLT-mediated regulation of mitochondrial function and redox balance are summarized in Table 3.

## 6. Functional Implications in Reproductive Systems

### 6.1. Oocyte Quality and Maturation

Oocyte quality is a key factor involved in female fertility and embryo development. Maturation of the oocyte involves an energy-demanding process that requires proper functioning and interaction of mitochondria, redox status, and the cytoskeleton [104]. Mitochondria in the oocytes provide ATP for the proper organization of the meiotic spindle apparatus, separation of chromosomes, and cytoplasmic maturation. Thus, low oocyte competence and developmental ability are associated with impaired mitochondrial activity [105].

Recently, there was evidence that MLT has protective functions in terms of maintaining oocyte quality by keeping mitochondrial functionality and preventing OS. Melatonin has been shown in studies to improve mitochondrial localization, increase mitochondrial membrane potential, and promote ATP generation in oocytes. Thus, MLT has been shown to support meiotic progression and reduce spindle abnormalities in experimental models [106,107,108,109,110].

Moreover, MLT reduces OS by preventing the excess generation of ROS and improving the antioxidant defense system. These characteristics are especially crucial when discussing aged oocytes, since these cells exhibit increased oxidative processes that reduce female fertility [111,112]. Consistent with these observations, experimental evidence has shown that melatonin may delay postovulatory oocyte aging and extend the window for successful fertilization, thereby preserving oocyte developmental competence [113].

### 6.2. Sperm Function and Male Fertility

The functionality of sperm depends heavily on mitochondrial metabolism, particularly ATP production, which drives sperm motility and fertilizing capacity. Mitochondria are localized in the sperm midpiece, where they generate the energy required for flagellar movement. Therefore, impairment of mitochondrial function may lead to reduced sperm motility and compromised male fertility [24,25,114].

MLT is known to have a beneficial effect on sperm cells by reducing OS and boosting mitochondrial metabolism. The excessive amount of reactive oxygen species in the sperm cells results in lipid peroxidation, DNA fragmentation, and poor motility. Sperm cells are particularly vulnerable to OS due to their tiny cytoplasmic volume and limited antioxidative activity [44,115].

Through its antioxidant effects and regulation of mitochondrial activity, MLT helps preserve the integrity of sperm membranes, mitochondria, and DNA. It is also thought to be involved in the regulation of essential processes such as sperm capacitation and the acrosome response [25,44,114].

### 6.3. Early Embryo Development and Viability

The embryonic phase becomes essential in terms of proper regulation of mitochondria and their activity. Immediately after an embryo is formed, the latter starts using the mitochondria provided by the mother for metabolic and dividing purposes [28,116,117].

Mitochondrial dysfunction during this time results in aberrant cell division, programmed cell death, and embryonic arrest. MLT can enhance the quality and survival of the embryo by preventing mitochondrial damage due to OS. It has been shown in studies both in vivo and in vitro that improved mitochondrial function and redox balance correlate with blastulation, more cells, and less apoptosis [118,119,120].

Moreover, MLT plays an important role in embryogenesis through the regulation of redox-sensitive signaling pathways [121].

### 6.4. Implications for ART

The impact of OS in constraining the effectiveness of ART, including IVF, has long been acknowledged. The culture medium, oxygen level, and laboratory manipulation may all lead to ROS generation, thereby influencing sperm or oocyte quality as well as embryo formation [122].

There is great potential in using MLT as an additive in the ART process because it has been shown that it can increase mitochondrial functionality while preventing OS. Both systemic MLT administration and supplementation of culture media with MLT have been associated with improvements in oocyte quality, embryonic development, and embryo survival rates [106,110,112]. Nevertheless, the existing literature on this subject is rather limited due to the inconsistent results and wide variation in experimental parameters.

However, its application could be beneficial in enhancing the outcomes of fertility treatment using ART techniques. A critical future task is the determination of the appropriate dose and time of administering MLT for achieving positive outcomes [123,124,125].

**Figure 2:**
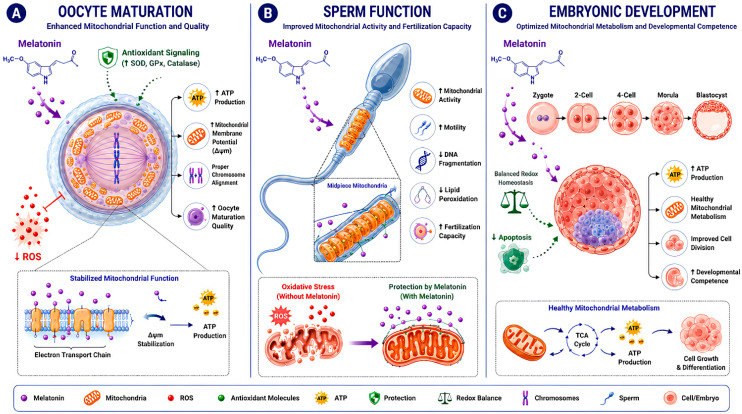
Effects of MLT on mitochondrial function and reproductive competence. (**A**) In oocytes, MLT enhances mitochondrial function, stabilizes mitochondrial membrane potential, reduces ROS levels, and supports chromosome alignment and oocyte maturation. (**B**) In sperm cells, MLT has been associated with improved mitochondrial function and motility, as well as reduced OS, DNA fragmentation, and lipid peroxidation, potentially contributing to enhanced fertilization capacity. (**C**) During embryonic development, MLT supports mitochondrial metabolism, ATP production, redox homeostasis, and reduced apoptosis, contributing to improved developmental competence and embryo viability.

In general, the experimental findings suggest that the physiological function of MLT is characterized by numerous roles in the reproductive system. MLT’s biological activity is linked to mitochondrial activity and cell metabolism [126,127,128]. The effects of MLT on mitochondrial function and redox homeostasis in reproductive cells and early embryos are summarized in Figure 2.

## 7. Clinical and Translational Evidence

### 7.1. Evidence from Human Studies

However, there is considerable interest in the potential role of MLT in reproductive medicine, particularly among infertile patients and individuals undergoing ART. Several clinical studies suggest that MLT supplementation may improve oocyte quality, fertilization rates, and embryo development, although evidence regarding major clinical outcomes, including live birth, remains limited and heterogeneous [112,123,124].

MLT has been shown to improve oocyte quality, fertilization rates, and embryo development in patients receiving IVF treatment [123,128]. These findings are supported by earlier experimental evidence demonstrating improved fertilization and embryo development following melatonin supplementation in mouse IVF models [129]. The mechanism behind this could be linked to decreased OS within the follicle, which is marked by reduced amounts of ROS and an increase in antioxidant levels in the follicular fluid [130]. Moreover, MLT treatment has also been reported to result in pregnancy success in certain studies. Nonetheless, it must be noted that most studies conducted are on a relatively small sample size and use diverse methodologies and reproductive outcomes [122,131,132].

MLT’s role in treating male infertility has been assessed according to its ability to positively affect sperm characteristics like sperm motility, concentration, and sperm DNA integrity [44,133]. According to some research, MLT can be beneficial to sperm characteristics, especially in high levels of OS. Nevertheless, evidence on this issue is still sparse and contradictory [115,134]. On the contrary, the therapeutic potential of MLT has been investigated extensively in women with reproductive disorders, particularly those undergoing ART and those with poor oocyte quality [135,136,137,138].

Several studies have shown encouraging results, but currently, there is not enough evidence for formulating treatment guidelines because of small sample size and different methodologies used. A comparative overview of representative clinical studies investigating melatonin supplementation in reproductive medicine, including study design, treatment protocols, major findings, and limitations, is presented in Table 4.

### 7.2. Evidence from Animal Models and Experimental Systems

Several animal studies along with in vitro experiments have offered experimental data proving the importance of MLT for the reproductive process. Most of the available evidence originates from mammalian models, particularly rodents, although other vertebrate species have also been investigated. The positive effects on oocyte maturation and embryogenesis along with protection from OS-associated damages have been observed in numerous animal species. It has been revealed that enhanced antioxidant properties and decreased levels of ROS can contribute to such beneficial outcomes [106,108,110,111,119,139].

Further evidence regarding the mechanisms involved in MLT-mediated reproductive protection includes the facilitation of antioxidant activities, maintenance of mitochondrial functioning, and control of apoptosis [15,45,94]. Further mechanisms that have been suggested based on experimental findings include changes in mitochondrial membrane potential, increased efficacy of the ETC, and stimulation of antioxidant defenses in the body [50,91,106]. Increased cell tolerance to OS, for instance, can be attributed to the stimulation of the Nrf2 pathway [64,66,84]. In addition to mammalian models, lower vertebrates such as fish have emerged as useful systems for studying melatonin biology. Recent evidence suggests that both melatonin and its metabolite AFMK contribute to antioxidant protection in rainbow trout eggs and ovarian fluid, supporting their role in redox homeostasis during reproduction and early developmental stages [139]. The applicability of experimental findings to human reproductive physiology is hampered by variations in reproductive functions across species and by laboratory restrictions [112,122,123].

### 7.3. Dosing, Timing, and Pharmacological Considerations

The most pressing challenge to the clinical application of MLT studies has been the inconsistent dosing and administration protocols used. Studies conducted on animals have involved highly variable doses, many above physiological limits, leaving one to question both the safety and effectiveness of MLT therapy [112,122,123,132].

MLT exhibits complex pharmacokinetic properties, including variable oral bioavailability and a relatively short half-life [4,74,77]. Therefore, timing may play a critical role in achieving a specific physiological effect of the hormone. Given that MLT functions as a circadian regulator, its administration should ideally mimic the body’s natural circadian pattern. Nevertheless, relatively few studies have systematically investigated the importance of administration timing in relation to the circadian phase [1,19,69,70].

Physiological nocturnal plasma melatonin concentrations generally occur in the picomolar-to-low nanomolar range. In contrast, many experimental and clinical studies employ pharmacological doses that produce circulating concentrations substantially exceeding endogenous levels. While physiological concentrations primarily support circadian synchronization and basal redox regulation, supraphysiological doses may exert additional receptor-independent antioxidant and mitochondrial effects [4,15,43,51]. Consequently, extrapolation of findings from high-dose experimental studies to physiological reproductive regulation should be undertaken cautiously.

In addition, pharmacological effects that differ from the physiological action of endogenous MLT may result from administration at doses higher than normal [62,67,81].

These limitations complicate comparisons between studies and may partly explain the inconsistencies observed between experimental and clinical findings [122,123,132].

### 7.4. Limitations and Current Gaps in the Literature

Although increasing attention has been paid to the therapeutic potential of MLT in reproductive medicine, several significant limitations remain. First, there is significant heterogeneity among studies regarding patient selection criteria, treatment regimens, and evaluation endpoints. As a result, direct comparison between studies and interpretation of the collective findings remain difficult [122,123,132].

Furthermore, randomized clinical trials in this field remain limited. A majority of these studies are conducted on a relatively smaller scale and not randomized as well, and the sample size of patients involved is not large enough [122,123,139]. Additionally, further research is also required to find out how different individual characteristics affect the treatment responses of MLT. Such characteristics include individual age, circadian rhythm, cause of infertility, and metabolism [19,69,112,126]. Another consideration that needs to be taken into account includes publication bias.

### 7.5. Translational Perspective and Therapeutic Potential

All things considered, it is evident that the obtained information provides substantial evidence about the possible involvement of MLT in regulation of reproductive physiology through its influence on mitochondrial metabolism and redox balance [15,23,43,127].

In this respect, special attention needs to be paid to elaborating recommendations for standardizing MLT therapy in terms of dosing and timing, and patient selection, in whom it will be effective. The approach based on taking into account the laws of the circadian rhythm might prove to be beneficial in this case [112,122,123,132].

Nonetheless, further mechanistic research on a larger scale is required to conclusively incorporate MLT into the scope of evidence-based reproductive medicine [69,70].

Overall, the combination of circadian rhythm, mitochondrial biology, and redox homeostasis offers an exciting prospect regarding the complex role of MLT in reproduction [23,69].

## 8. Conceptual Integration and Future Perspectives

### 8.1. Integrative Model Linking Circadian Signaling, Mitochondrial Function, and Reproduction

In light of the above-presented data, the classical assumption that MLT serves only as an antioxidant and a circadian signaling molecule is not enough to provide a full description of its physiological roles [9,140]. In this regard, it may be regarded as a signal mediator connecting the circadian system with mitochondria in reproductive cells [126].

Within this framework, circadian rhythms regulate MLT secretion, while MLT modulates mitochondrial activity and redox homeostasis through interconnected processes including oxidative phosphorylation, ROS regulation, and apoptosis [141]. Consequently, mitochondria emerge as central integrators of circadian and reproductive signaling. Emerging evidence also suggests interactions between MLT signaling and key components of the circadian clock, including the CLOCK, BMAL1, PER, and CRY pathways, which may further influence mitochondrial metabolism and reproductive physiology [69,142,143].

Therefore, MLT exerts multiple biological effects, including enhancing mitochondrial efficiency, stabilizing mitochondrial membrane potential, regulating mitochondrial dynamics, and maintaining redox homeostasis through ROS scavenging and the activation of endogenous antioxidant systems [5].

Additionally, this theory emphasizes the importance of timing. An alteration in the circadian rhythm may interfere with the synchronization between the MLT signaling pathway and mitochondrial function, leading to poor energy metabolism, increased generation of ROS, and poor reproductive success. In this sense, MLT can be considered a protective mediator that helps preserve mitochondrial balance amid altered circadian rhythms [125,140].

### 8.2. Implications for Reproductive Medicine and Chronobiology

The use of circadian biology in reproductive medicine is a relatively new area with great therapeutic promise. In this context, proper regulation of MLT pathways can benefit reproductive health, especially in cases involving oxidative damage and mitochondrial impairment [7,15,71].

In clinical practice, aligning ART procedures with circadian rhythms, including the timing of oocyte retrieval, hormone administration, and embryo culture conditions, can improve reproductive outcomes. Furthermore, MLT administration may help improve mitochondrial function and reduce OS within the reproductive microenvironment. These observations support the emerging concept of chronobiologically guided reproductive medicine, in which temporal synchronization may represent an additional therapeutic dimension in fertility treatment [10,15,122].

In general, reproduction is dependent on the healthy functioning of the circadian rhythm. Disturbance of the circadian rhythm due to shift work, lack of sleep, and too much artificial light may have an adverse impact on reproductive physiology via modifications in MLT levels, mitochondrial activity, and redox state [7,69,140].

### 8.3. Future Research Directions

Despite much achieved in terms of understanding the biological activity of MTL, there are still many aspects of its action that require further investigation. It is necessary to gain insight into the mechanisms of MLT-induced mitochondria function regulation in various reproductive cells. A special focus needs to be made on the role of signaling cascades in mitochondrial adaptation and redox regulation, particularly on Nrf2-dependent regulation mechanisms [15,16,84].

Importantly, the strength of evidence supporting the proposed mechanisms is not uniform. While some effects of MLT on reproductive outcomes have been evaluated in human and ART studies, many mechanistic pathways, including Nrf2 signaling, SIRT3 regulation, mitochondrial bioenergetics, and receptor-independent actions, have been investigated predominantly in animal models and in vitro systems. Therefore, further translational studies are needed to validate these mechanisms in human reproductive tissues and clinical settings [71,122,132].

Another area of interest is connected with establishing standard approaches to MLT delivery. The optimal dosing regime is crucial to achieve positive outcomes, and thus, should be defined accurately. Individual peculiarities associated with the circadian phenotype and reproductive conditions have to be considered when developing the appropriate protocol [71,122,132].

Lastly, proper randomized controlled trials should be conducted to establish the effectiveness and safety of MLT-based therapy in reproductive medicine [119,120,126].

In conclusion, integrated models involving the circadian system, mitochondrial biology, and reproductive function may offer insights into the mechanisms behind reproductive function and help develop therapies for disorders in reproductive function [18,69,135].

## 9. Conclusions

MLT has traditionally been regarded as a circadian hormone and antioxidant. However, accumulating evidence suggests that it also participates in the regulation of mitochondria homeostasis and reproductive biology. Experimental and clinical studies indicate that MLT may modulate mitochondrial metabolism, OS, and antioxidant signaling pathways, including Nrf2-mediated mechanisms, potentially contributing to improved gamete quality, fertilization, and embryo development.

Importantly, MLT may serve as a biological link between circadian signaling and mitochondrial regulation, enabling the integration of environmental timing cues with reproductive physiology. While encouraging findings have been reported in experimental models and clinical studies, the strength of evidence varies across outcomes. Beneficial effects on OS regulation, mitochondrial function, and laboratory ART outcomes appear relatively consistent, whereas evidence regarding major clinical endpoints, particularly live birth rates, remains limited and heterogeneous. Furthermore, variability in study design, patient populations, dosing regimens, and treatment timing continues to hinder clinical translation.

In summary, MLT represents a promising circadian-driven mediator of mitochondrial biology and redox homeostasis, with potential relevance to reproductive medicine. Nevertheless, further well-designed mechanistic studies and adequately powered clinical trials are required to clarify its therapeutic role and establish evidence-based recommendations for clinical practice.

## Figures and Tables

**Table 1:** Roles of mitochondrial function and redox signaling in reproductive cells and early embryonic development.

Reproductive Cell Type	Mitochondrial Functions	Role in Reproductive Physiology	Consequences of Mitochondrial Dysfunction
Oocyte [20,21,40]	ATP production for meiotic maturation; regulation of spindle formation and cytoplasmic maturation	Supports oocyte maturation and intracellular signaling	Impaired spindle formation, mitochondrial dysfunction, reduced developmental competence
Sperm [24,25,35]	ATP production for motility (midpiece mitochondria); regulation of capacitation	Required for capacitation and acrosome reaction	Lipid peroxidation, DNA fragmentation, reduced motility and fertilization capacity
Early Embryo [22,28,37]	Energy supply for cell division and developmental progression; regulation of apoptosis	Modulates signaling pathways for proliferation and differentiation	Developmental arrest, increased apoptosis, altered gene expression

**Table 2:** Physiological effects of melatonin on mitochondrial function and redox homeostasis.

Process	Target/Process	Effect of MLT	Functional Outcome in Reproductive Cells
Mitochondrial bioenergetics [15,48,50]	ETC, ATP production	Enhances ETC efficiency, reduces electron leakage, preserves membrane potential	Increased ATP production; improved oocyte maturation, sperm motility, and embryo development
Mitochondrial dynamics [43,52,55]	Fusion and fission processes	Modulates expression and activity of fusion–fission proteins	Maintenance of mitochondrial integrity and proper distribution
Apoptotic regulation [57,58,59]	Bcl-2/Bax balance, cytochrome c release	Enhances anti-apoptotic signaling and stabilizes mitochondrial membranes	Reduced apoptosis and improved gamete and embryo viability
Direct antioxidant activity [15,61,62]	ROS	Scavenges free radicals (e.g., hydroxyl radicals, superoxide anions)	Reduced oxidative damage
Indirect antioxidant regulation [16,65,66]	Nrf2 signaling pathway	Activates antioxidant enzymes (SOD, GPx, catalase)	Enhanced cellular antioxidant capacity
Circadian regulation [18,69,70]	Mitochondrial metabolic rhythms	Synchronizes mitochondrial activity with circadian signals	Optimized energy metabolism and redox balance

**Table 3:** Molecular pathways underlying melatonin-mediated mitochondrial regulation.

Mechanism	Target/Pathway	Effect of MLT	Functional Outcome in Reproductive Cells
Receptor-dependent signaling	MT1/MT2 receptors, cAMP, protein kinases	Modulates intracellular signaling pathways and gene expression	Regulation of cellular metabolism and survival [74,77,103]
Receptor-independent action	Mitochondrial membranes, ETC	Direct mitochondrial accumulation; stabilization of mitochondrial membranes; antioxidant activity	Protection against oxidative damage at the mitochondrial level [15,43,51]
Antioxidant pathway activation	Nrf2 signaling pathway	Upregulation of antioxidant enzymes (SOD, GPx, catalase)	Enhanced redox homeostasis and mitochondrial integrity [64,65,66]
Bioenergetic regulation	ETC complexes I and IV, oxidative phosphorylation	Increased efficiency of electron transport and ATP production; reduced electron leakage	Improved energy supply for gametes and embryos [48,50,91]
SIRT3 activation	Mitochondrial deacetylase SIRT3	Deacetylation and activation of metabolic and antioxidant enzymes	Enhanced mitochondrial function and reduced OS [93,94,95]
Apoptotic regulation	Bcl-2/Bax balance, cytochrome c release, mitochondrial permeability transition pore	Increased anti-apoptotic signaling; inhibition of mitochondrial membrane permeabilization	Reduced apoptosis and improved cell survival [79,96,98]

**Table 4:** Summary of clinical studies evaluating melatonin supplementation in reproductive medicine.

Study	Study Design	Population	Melatonin Regimen	Main Findings	Limitations
Hu et al. [122]	Systematic review and meta-analysis of randomized trials	Women undergoing ART	Variable protocols and dosages	Increased clinical pregnancy rate, retrieved oocytes, MII oocytes, and good-quality embryos; no significant improvement in live birth	Low-quality evidence, heterogeneous populations, limited live birth data
Wu et al. [123]	Systematic review and meta-analysis of randomized trials	Women undergoing ART; 11 RCTs, *n* = 1481	Variable protocols and dosages	Increased clinical pregnancy rate, fertilization rate, MII oocytes, and high-quality embryos; no significant improvement in oocyte yield	Moderate-to-high heterogeneity, possible publication bias, lack of standardized protocols
Sadeghpour et al. [124]	RCT	Women with diminished ovarian reserve undergoing ART (*n* = 68)	3 mg/day melatonin from day 5 of menstrual cycle before ovarian stimulation	Increased retrieved oocytes, fertilization rate, embryo quality, biochemical pregnancy rate, rGSH, and TAC	Small sample size, single-center study, no live birth data
Veiga et al. [128]	Systematic review and meta-analysis	Women undergoing ART	Variable protocols and dosages	Improved fertilization rate, MII oocytes, and antral follicle count; no significant improvement in clinical pregnancy	Heterogeneity among studies, variable protocols, limited evidence for major clinical endpoints
Bao et al. [131]	Prospective clinical trial	Patients with repeated poor-quality embryos and vitrified-warmed embryos	Melatonin 10^−7^ M added to embryo culture medium	Increased Day 3 high-quality embryo rate and blastocyst formation; trend toward higher clinical pregnancy; upregulated CAT expression	Small sample size, single-center design, no significant improvement in major clinical outcomes
Tang et al. [132]	Systematic review and meta-analysis	Women undergoing ART; 11 studies	Variable protocols and dosages	Improved fertilization rate, MII oocytes, and top-quality embryos; no significant improvement in live birth	Heterogeneous populations, limited live birth data, insufficient evidence for routine clinical use
Takasaki et al. [135]	Prospective clinical study	Women with previous poor fertilization outcomes undergoing IVF (*n* = 27)	Oral melatonin, 1 or 3 mg nightly from day 5 of the previous menstrual cycle until hCG administration	Increased intrafollicular melatonin, reduced oocyte degeneration, improved follicular oxidative balance, and trend toward increased fertilization	Small sample size, non-randomized design, historical controls, limited pregnancy data
Espino et al. [138]	Randomized pilot study	Women with unexplained infertility undergoing IVF	Oral melatonin, 3 or 6 mg/day from ovarian stimulation until follicular puncture	Improved follicular oxidative balance and oocyte quality; slight increase in pregnancy/live birth rates	Pilot design, small sample size, limited statistical power

## Data Availability

No new data were created or analyzed in this study.

## Abbreviations

The following abbreviations are used in this manuscript:

MLT	Melatonin
ROS	Reactive oxygen species
OS	Oxidative stress
IVF	In vitro fertilization
ART	Assisted reproductive technologies
ETC	Electron transport chain
